# Les syringomes

**DOI:** 10.11604/pamj.2019.32.194.16161

**Published:** 2019-04-18

**Authors:** Fatima-zahra Agharbi

**Affiliations:** 1 Hôpital Civil Tétouan, Tétouan, Maroc

**Keywords:** Syringomes, tumeurs, annexiels, syringomas, tumors, adnexal

## Abstract

Syringomas are benign tumors of cutaneous appendages of eccrine or apocrine origin affecting approximately 1% of the population. They mainly occur in women and they commonly manifest as soft skin-colored or slightly yellowish papules on the lower eyelid and the upper part of the cheeks. More rarely, syringomas can even occur on the neck, the armpits, the breasts, the lower portion of the abdomen, the thighs and the groin. Clinically, they can be distinguished from xanthelasmas, warts or cancers because they are monomorphic and have a regular distribution. In doubtful cases, the diagnosis is confirmed by biopsy. Syringomas can be easily detected on histological examination due to the presence of comma-shaped sweat ducts in the dermis. Even though syringomas are benign tumors, their appearance can be embarrassing to the patients. Therapeutic options, mainly supported by small case series and case reports, include surgical excision, electrodessication, curettage, chemical exfoliation, cryosurgery and laser treatment. However, as these tumors lie deep in the dermis, all treatments are associated with a substantial risk of recurrence and can cause scars and skin pigment changes. We here report the case of a 40-year old woman with no previous history, presenting with skin-colored periorbital papules whose histological examination showed syringomas.

## Image en médecine

Les syringomes sont des tumeurs bénignes des annexes cutanées qui tireraient leur origine des canaux exocrines ou apocrines et qui affectent environ 1% de la population. Ils s'observent le plus souvent chez les femmes et prennent généralement la forme de papules molles de couleur chair ou légèrement jaunâtres à la paupière inférieure et à la partie supérieure des joues. Plus rarement, les syringomes s'observent ailleurs qu'au pourtour des yeux, soit au cou, aux aisselles, aux seins, à la portion inférieure de l'abdomen, aux cuisses et à l'aine. Au plan clinique, les syringomes se distinguent des xanthélasmas, des verrues ou des cancers par leur caractère monomorphe et leur distribution régulière; le diagnostic est confirmé par biopsie dans les cas douteux. À l'examen histologique, les syringomes se reconnaissent facilement par la présence de canaux sudoripares ayant la forme de virgules dans le derme. Même si les syringomes sont bénins, leur apparence peut être gênante pour les patients. Les options thérapeutiques, étayées pour la plupart par de petites séries et rapports de cas, incluent l'excision chirurgicale, l'électrodessication et curetage, l'exfoliation chimique, la cryochirurgie et le traitement au laser. Toutefois, comme ces tumeurs sont profondément ancrées dans le derme, tous les traitements sont associés à un risque substantiel de récurrence et peuvent entraîner des cicatrices et des changements pigmentaires. Nous rapportons l'observation d'une patiente de 40 ans sans antécédents qui présentait des papules couleur chair péri-orbitaires dont l'étude histologique était en faveur de syringomes.

**Figure 1:**
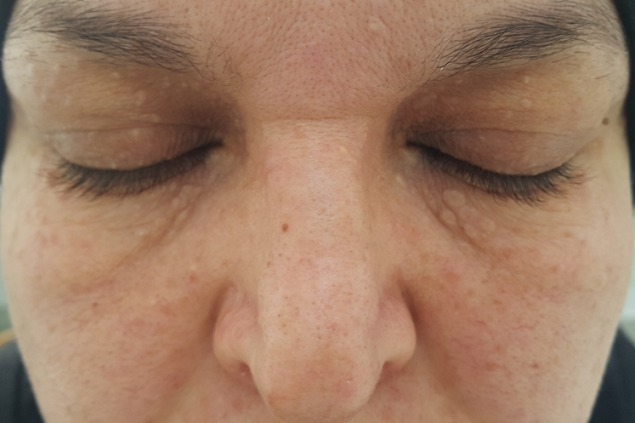
Papules couleur chair péri-orbitaires

